# YAP1 is a Prognostic Biomarker and Correlated with Immune Cell Infiltration in Pancreatic Cancer

**DOI:** 10.3389/fmolb.2021.625731

**Published:** 2021-06-02

**Authors:** Kai Sun, Xue-de Zhang, Xiao-yang Liu, Pei Lu

**Affiliations:** ^1^ Department of Oncology, Liuzhou People’s Hospital, Liuzhou, China; ^2^ Department of Hematology and Oncology, Beilun District People’s Hospital, Ningbo, China; ^3^ Department of General Surgery, People's Hospital of Gansu Province, Lanzhou, China

**Keywords:** pancreatic cancer, Hippo pathway, prognosis, Yes-associated protein-1, tumor infiltration

## Abstract

Yes-associated protein-1 (YAP1) is an important effector of the Hippo pathway and has crosstalk with other cancer signaling pathways. It induces an immunosuppressive tumor microenvironment by activating pathways in several cellular components. However, the mechanisms by which it drives immune infiltration in pancreatic cancer remain poorly understood. We analyzed the expression of YAP1 as well as its prognostic value and correlations with immune infiltrates in various cancers, with a focus on pancreatic cancer. In particular, using the Oncomine database and Gene Expression Profiling Interactive Analysis (GEPIA) database, we found that YAP1 is differentially expressed between tumor tissues and control tissues in a number of cancers and in particular, is elevated in pancreatic cancer. Using the Kaplan–Meier plotter, GEPIA, and Long-term Outcome and Gene Expression Profiling database of pan-cancers (LOGpc), we further established the prognostic value of YAP1. We found that YAP1 expression was significantly related to outcomes in multiple types of cancer based on data from The Cancer Genome Atlas, particularly in pancreatic cancer. Correlations between YAP1 and immune cell infiltration and immune cell marker expression were examined using Tumor Immune Estimation Resource and GEPIA. High expression levels of YAP1 were significantly associated with a variety of immune markers and immune cell subsets in pancreatic cancer. These results suggest that YAP1 is correlated with patient outcomes and tumor immune cell infiltration in multiple cancer types and is a valuable prognostic biomarker in pancreatic cancer.

## Introduction

Pancreatic cancer is the fifth most common cause of cancer-related deaths in developed countries, accounting for 260,000 deaths worldwide every year; its 5-years survival rate is extremely low (5%) (Johansson et al., 2016). Because the early detection of pancreatic tumors is relatively difficult, only as few as 20% of patients are eligible for possible radical surgery; in addition, chemotherapy and radiotherapy do not substantially improve overall survival (OS) (Martinez-Bosch et al., 2018). However, since immunotherapy was declared a breakthrough approach in 2013, the effectiveness of immune checkpoint inhibition has been demonstrated in various solid tumors, and it may be beneficial in pancreatic cancer (Gan et al., 2020). Clinical studies of immunotherapy for pancreatic cancer are ongoing (Zhang and Choi, 2015; Martinez-Bosch et al., 2018), and the characterization of immunophenotypes and identification of novel immune-related therapeutic targets in pancreatic cancer are urgent research goals (Luedke et al., 2012).

Yes-associated protein-1 (YAP1) is a transcriptional coactivator and is a pivotal factor in the Hippo/YAP signaling pathway, promoting tumor formation and development (Zanconato et al., 2019; Kuenzi and Ideker, 2020). YAP1 expression is significantly correlated with the expression levels of several proto-oncogenes, such as *KRAS*, *Wnt/*β*-catenin*, and *CTGF* (Nguyen and Yi, 2019; Pobbati and Hong, 2020). The expression of YAP1 is elevated in a number of cancer types, such as liver, lung, colorectal, ovarian, and prostate cancers. (Sharma et al., 2017; Yamauchi and Moroishi, 2019). Dephosphorylated YAP1 accumulates in the nucleus due to the inactivation of Hippo signaling, thereby affecting cell proliferation, invasion, epithelial-mesenchymal transition, stemness, and metabolic reprogramming (Zhang et al., 2017; Flinn et al., 2019; Liu et al., 2019; Meng et al., 2020). YAP1 in cancer cells also confers resistance to certain drugs (Zhou et al., 2018; Yao et al., 2019).

Studies of YAP1 in tumor immunity are in the early stages. Early studies have shown that the excessive activation of YAP and TAZ inhibits tumor growth via TEAD-mediated transcription (Gebhardt and Harvey, 2016; Moroishi et al., 2016). Another study has indicated that YAP1 regulates innate immunity by interacting with IRF3 (Du et al., 2018). There is evidence for important roles of YAP in the regulation of the tumor immune checkpoint PD-L1/PD-1 pathway in malignant pleural mesothelioma and non-small cell lung cancer (Miao et al., 2017; Hsu et al., 2018). These previous findings indicate that YAP1 may be a valuable prognostic biomarker (Kim et al., 2018). Although pancreatic cancer is relatively immune-resistant due to tissue fibrosis in the tumor microenvironment and a lack of TILs, B and T cell-specific immune responses are still produced under exposure to tumor cell antigens (Zhang and Choi, 2015; Ibrahim and Wang, 2016). Therefore, the mechanisms by which YAP1 functions in the immune microenvironment in pancreatic cancer deserve further study.

In this study, we systematically investigated YAP1 expression and its relationship with prognosis based on LOGpc, Oncomine, GEPIA, and K-M plotter analyses. Next, we analyzed the correlations between YAP1 and tumor-infiltrating immune cells in the microenvironments of several tumors using TIMER. Our results suggest that YAP1 expression is related to pancreatic cancer and the immune microenvironment.

## Materials and Methods

### Oncomine Database Analysis

Pan-cancer gene expression array data from the ONCOMINE database (www.oncomine.org), including expression data for 715 genes in 86,733 samples, were obtained. The Student’s *t*-test was used to compare the mRNA expression levels of *YAP1* between cancer specimens and normal specimens. The cut-off *p-*value was 0.01, and the threshold fold change was 2.0.

### Prognostic Value of YAP1 Expression

The relationship between the expression of YAP1 and prognosis was evaluated using two databases, **t**he Long-term Outcome and Gene Expression Profiling database of pan-cancers (LOGpc) (http://bioinfo.henu.edu.cn/DatabaseList.jsp) and Kaplan–Meier plotter (KM plotter; http://kmplot.com/analysis/) (Győrffy et al., 2013). LOGpc included 209 expression datasets and 13 survival analyses of 27 distinct malignancies involving 31,310 patients. Kaplan–Meier plotter is an online database of microarray gene expression data and survival information derived from European Genome-Phenome Archive, Gene Expression Omnibus, and TCGA, including data for 21 different types of cancers and a large number of samples for different cancers cohorts (Györffy et al., 2010).

### TIMER Database Analysis

The TIMER (https://cistrome.shinyapps.io/timer/) database was used to explore immune cell infiltration in various cancers. Data for tumor immune infiltration [B cells, CD4^+^ T cells, CD8^+^ T cells, neutrophils, macrophages, and dendritic cells (DCs)] determined by statistical methods and validated by pathological examinations are included. Specific immune cell subsets were used to explore the relationship between YAP1 expression and the degree of infiltration. A KM survival analysis was performed to explore the relationship between survival and gene expression or immune cell infiltration. Finally, associations between YAP1 expression and the expression of markers of specific infiltrating immune cell subsets were evaluated.

### GEPIA Database Analysis

Gene Expression Profiling Interactive Analysis (GEPIA) uses standard processing pipelines to analyze RNA sequencing expression data for 8,587 normal samples and 9,736 tumors from GTEx and TCGA projects. The relationships between YAP1 expression levels and patient prognosis in several cancers and the link between YAP1 expression and immune cell infiltration, with a focus on tumor markers, were evaluated.

### Statistical Analysis

Data were analyzed with a log-rank test, and the HR, fold-change, and *p-*values were obtained. Spearman's correlation analysis was used to measure the degree of relationship between specific variables, where correlation coefficients *r* indicate the strength of the relationship as follows: very weak, 0.00–0.19; weak, 0.20–0.39; moderate, 0.40–0.59; strong, 0.60–0.79; very strong, 0.80–1.0. *p* < 0.05 was the threshold for significance.

## Results

### Assessment of YAP Expression in Cancer and Normal Tissues

**FIGURE 1:**
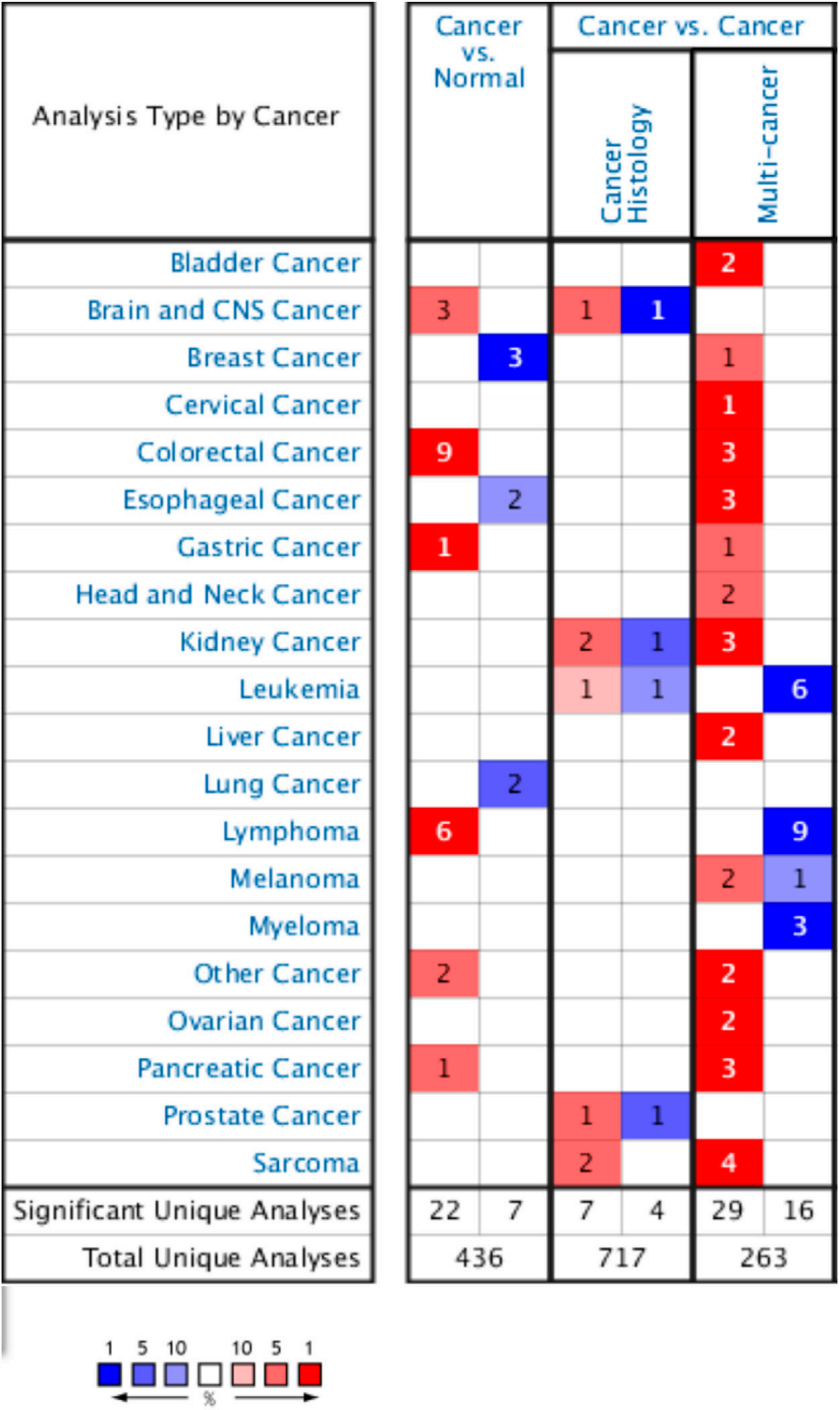
Transcript levels of YAP1 in different cancers based on the ONCOMINE database.

**FIGURE 2:**
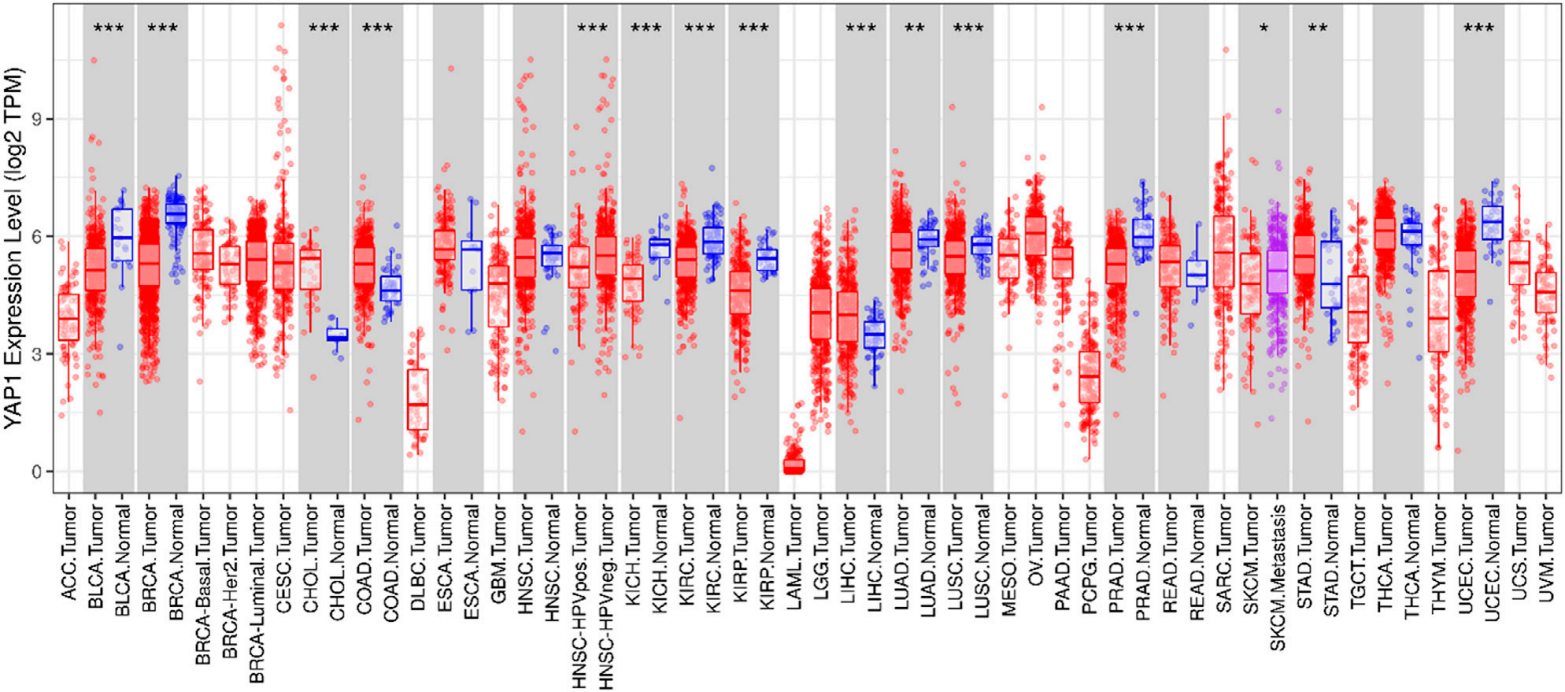
YAP1 expression levels in different tumor types based on data from TCGA were determined using TIMER. *p < 0.05, **p < 0.01, ***p < 0.001.

**FIGURE 3:**
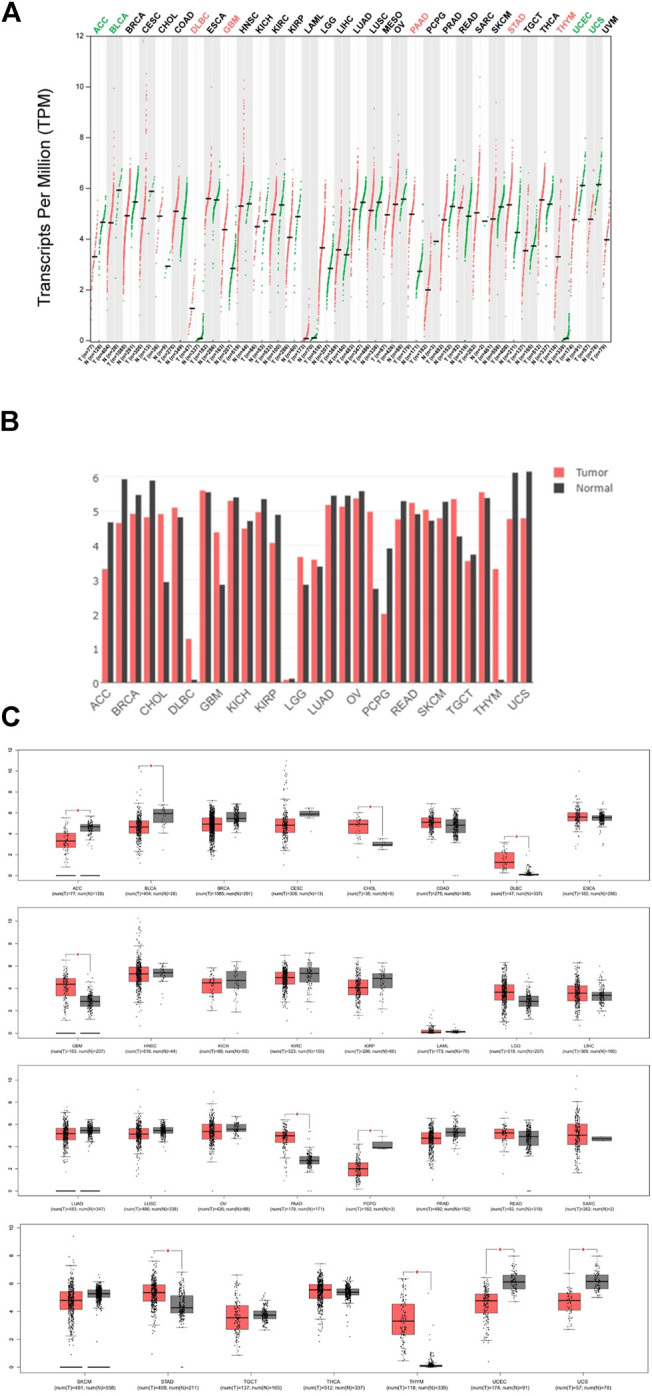
Expression of YAP1 in different cancers (GEPIA). **(A)** YAP1 expression profile across all tumor samples and paired normal tissues (dot plot). **(B)** YAP1 expression profile across all tumor samples and paired normal tissues (Bar plot). **(C)** YAP1 expression profile across all tumor samples and paired normal tissues (box plots).

The Oncomine database was used to compare YAP1 expression in pan-tumors and corresponding normal tissues. The expression levels of YAP1 were higher in tumor tissues than in normal control tissues for pancreatic, gastric, colorectal, and brain cancers and lymphoma. In breast, esophageal, and lung cancer tissues, YAP1 expression was lower than that in normal tissue controls (Figure 1). Table 1 summarizes the detailed findings for specific tumor types. Using the TCGA and TIMER databases, YAP1 expression was significantly lower in bladder urothelial carcinoma (BLCA), breast invasive carcinoma (BRCA), kidney chromophobe (KICH), kidney renal clear cell carcinoma (KIRC), kidney renal papillary cell carcinoma (KIRP), liver hepatocellular carcinoma (LIHC), lung adenocarcinoma (LUAD), lung squamous cell carcinoma (LUSC), prostate adenocarcinoma (PRAD), and uterine corpus endometrial carcinoma (UCEC) than in adjacent normal tissues. However, YAP1 expression was significantly higher in cholangial carcinoma (CHOL), colon adenocarcinoma (COAD), and stomach adenocarcinoma (STAD) than in adjacent normal tissues. Differences between YAP1 expression in tumors and normal tissues are summarized in Figure 2. Using GEPIA databases, the expression of YAP1 was significantly higher in tumor tissues than in normal controls in CHOL, Lymphoid Neoplasm Diffuse Large B-cell Lymphoma (DLBC), Glioblastoma multiforme (GBM), Pancreatic adenocarcinoma (PAAD), STAD, and Thymoma (THYM). In contrast, the expression of *YAP1* was significantly lower in tumor tissues than in normal control tissues in Adrenocortical carcinoma (ACC), BLCA, Pheochromocytoma and Paraganglioma (PCPG), uterine corpus endometrial carcinoma (UCEC), and Uterine Carcinosarcoma (UCS). Differences between the expression of YAP1 in tumors and normal adjacent tissue samples are shown in Figure 3.

**TABLE 1:** Differences in YAP1 expression between cancer tissues and normal tissues based on the Oncomine database.

*Cancer*	Cancer type	*p*-value	Fold change	Rank (%)	Sample	References
Brain and CNS	Glioblastoma vs. Normal	4.43E−12	18.906	2	230	Murat brain
	Glioblastoma vs. Normal	3.08E−14	2.265	3	579	Sun Brain
	Glioblastoma vs. Normal	4.43E−6	2.152	5	703	Bredel brain 2
Breast	Ductal breast carcinoma vs. Normal	1.31E−11	−2.796	1	110	Richardson breast 2
	Mucinous breast carcinoma vs. Normal	2.23E−5	−2.740	3	429	TCGA breast
	Invasive breast carcinoma stroma vs. Normal	6.32E−25	−8.170	4	667	Finak breast
Colorectal	Colon adenoma vs. Normal	1.24E−9	3.268	1	72	Skrzypczak colorectal 2
	Colon adenoma epithelia vs. Normal	2.35E−7	2.633	2	344	Skrzypczak colorectal 2
	Colon carcinoma vs. Normal	1.64E−6	2.120	10	1783	Skrzypczak colorectal 2
	Cecum adenocarcinoma vs. Normal	8.18E−9	2.238	1	144	Kaiser colon
	Rectal adenocarcinoma vs. Normal	6.69E−6	2.014	1	155	Kaiser colon
	Colon adenocarcinoma vs. Normal	2.89E−12	2.234	1	173	Kaiser colon
	Rectosigmoid adenocarcinoma vs. Normal	5.25E−6	2.158	3	394	Kaiser colon
	Rectal adenoma vs. Normal	2.19E−8	2.152	2	196	Sabates–Bellver Colon
	Colorectal carcinoma vs. Normal	9.10E−14	2.129	2	278	Hong Colorectal
Esophageal	Barrett's esophagus vs. Normal	4.04E−10	−3.347	6	967	Kim esophagus
	Esophageal adenocarcinoma vs. Normal	1.26E−11	−2.459	9	1,575	Kim esophagus
Gastric	Gastric intestinal type adenocarcinoma vs. Normal	2.22E−12	2.204	1	168	DErrico gastric
Lung	Small cell lung carcinoma vs. Normal	1.01E−5	−3.733	3	200	Bhattacharjee Lung
	Lung carcinoid tumor vs. Normal	2.68E−8	−8.584	5	373	Bhattacharjee lung
Lymphoma	Follicular lymphoma vs. Normal	1.16E−33	14.570	1	47	Compagno lymphoma
	Diffuse large B-Cell lymphoma vs. Normal	6.15E−23	10.179	2	215	Compagno lymphoma
	Germinal center B-cell-like diffuse large B-Cell lymphoma vs. Normal	1.02E−6	3.857	2	385	Compagno lymphoma
	Activated B-cell-like diffuse large B-Cell lymphoma vs. Normal	2.37E−9	6.351	5	799	Compagno lymphoma
	Unspecified peripheral T-Cell lymphoma vs. Normal	3.31E−14	10.931	2	357	Piccaluga lymphoma
	Angioimmunoblastic T-Cell lymphoma vs. Normal	3.67E−5	20.196	8	1,563	Piccaluga lymphoma
Others	Teratoma, NOS vs. Normal	7.73E−9	2.771	3	420	Korkola seminoma
	Yolk sac tumor, NOS vs. Normal	5.18E−6	3.674	4	649	Korkola seminoma
Pancreatic	Pancreatic ductal adenocarcinoma vs. Normal	9.82E−11	2.180	3	587	Badea pancreas

### Correlation Between the Expression of YAP1 and Prognosis

**FIGURE 4:**
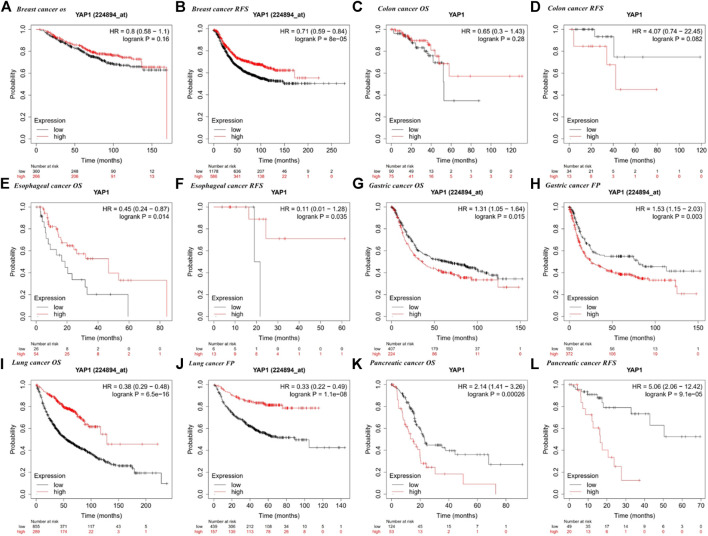
Kaplan–Meier survival curves for high and low YAP1 expression in different types of cancer based on the Kaplan–Meier plotter database. OS, overall survival; DFS, disease-free survival; RFS, relapse-free survival; DSS, disease-specific survival. DMFS, distant metastasis-free survival. FP, first progression.

We employed the KM plotter database to explore the effect of YAP1 expression on the survival of patients with cancers showing the most obvious expression differences between tumor tissues and normal tissues (i.e., breast, colorectal, esophageal, gastric, lung, and pancreatic cancers). For multiple cancer types, such as lung, esophageal, gastric, pancreatic, and breast cancer, we detected a significant correlation between prognosis and YAP1 expression (Figure 4). We revealed that a higher YAP1 expression level was significantly related to a poorer prognosis in pancreatic cancer (OS, HR = 2.14, 95% CI = 1.41–3.26, *p* = 0.00026; relapse free survival (RFS), HR = 5.06, 95% CI = 2.06–12.42, *p* = 9.1e−0.5) and gastric cancer (OS, HR = 1.31, 95% CI = 1.05–1.64, *p* = 0.015; first progression (FP), HR = 1.53, 95% CI = 1.15–2.03, *p* = 0.003) (Figures 4G,H,K,L). Increased YAP1 expression was associated with an improved prognosis in lung cancer (OS, HR = 0.38, 95% CI = 0.29–0.48, *p* = 6.5e−16; FP, HR = 0.33, 95% CI = 0.22–0.49, *p* = 1.1e−08) and esophageal cancer (OS, HR = 0.45, 95% CI = 0.24–0.87, *p* = 0.014; RFS, HR = 0.11, 95% CI = 0.01–1.28, *p* = 0.035) and with an improved RFS in breast cancer (RFS, HR = 0.71, 95% CI = 0.59–0.84, *P* = 8e−5) (Figures 4A,B,E,F,I,J). There was no significant correlation between the expression of YAP1 and prognosis in colorectal cancer.

**FIGURE 5:**
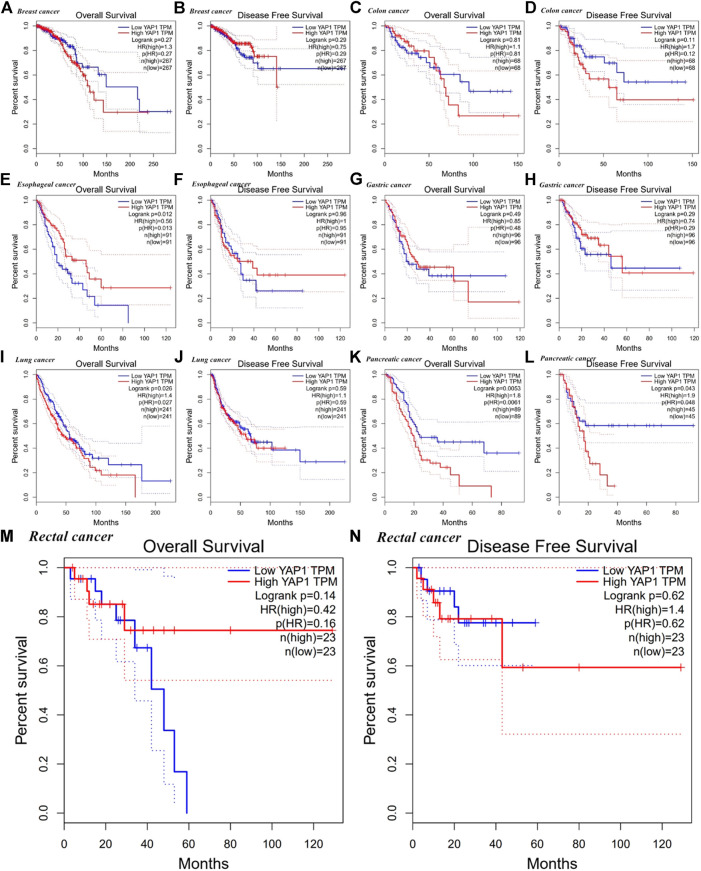
Kaplan–Meier survival curves for high and low YAP1 expression in different types of cancer based on GEPIA. OS, overall survival; DFS, disease-free survival; RFS, relapse-free survival; DSS, disease-specific survival. DMFS, distant metastasis-free survival.

Using the GEPIA database, we found that high YAP1 expression levels were associated with a poorer prognosis based on OS and disease-free survival (DFS) in pancreatic cancer (OS, HR = 1.8, *p* = 0.0053; DFS HR = 1.9, *p* = 0.043) but not in gastric cancer (OS, HR = 0.85, *p* = 0.49; DFS, HR = 0.74, *p* = 0.29) (Figure 5K,L,G,H). High expression of YAP1 was associated with a better OS in esophageal cancer (OS, HR = 0.56, *p* = 0.012) (Figure 5E). However, high mRNA levels of YAP1 were significantly correlated with a reduced OS in lung cancer (OS, HR = 1.4, *p* = 0.026) (Figure 5I). The expression of YAP1 was not significantly correlated with OS and DFS in breast, colorectal, and gastric cancers (Figures 5A–D,G,H).

**FIGURE 6:**
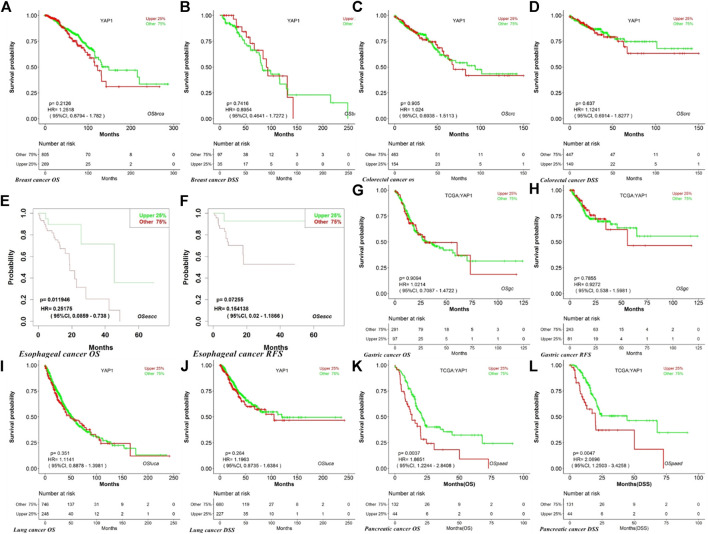
Kaplan–Meier survival curves for high and low YAP1 expression in different types of cancer based on the LOGpc TCCG database. OS, overall survival; DFS, disease-free survival; RFS, relapse-free survival; DSS, disease-specific survival. DMFS, distant metastasis-free survival.

We next used LOGpc to explore the link between YAP1 expression and outcomes for patients with breast, colorectal, esophageal, gastric, lung, and pancreatic cancers. TCGC database showed that higher YAP1 expression is linked to a poorer prognosis in pancreatic cancer (OS, HR = 1.87, 95% CI = 1.22–2.84, *p* = 0.0037; disease-specific survival (DSS) HR = 2.07, 95% CI = 1.25–3.43, *p* = 0.0047) (Figure 6). However, YAP1 expression was not significantly related to prognosis in breast, lung, gastric, esophageal, and colorectal cancer (Figures 6A–N). Our findings suggested that YAP1 has prognostic value in several types of cancers, especially pancreatic cancer, and the YAP1 expression patterns and prognostic value differed among cancer types.

### Association Between YAP1 Expression and Immune Cell Infiltration

**FIGURE 7:**
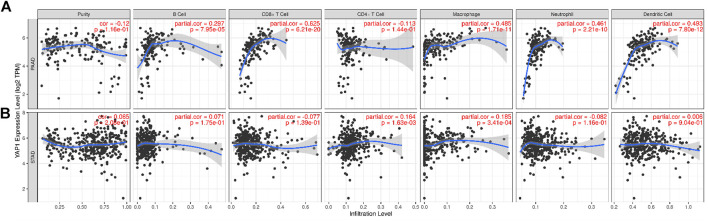
Correlation between YAP1 expression and immune cell infiltration in pancreatic adenocarcinoma (PAAD) and stomach adenocarcinoma (STAD).

**FIGURE 8:**
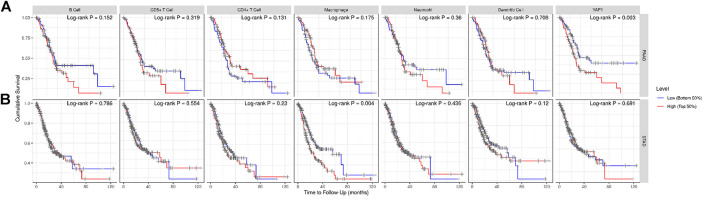
Kaplan–Meier plots of immune infiltration and YAP1 expression levels in pancreatic adenocarcinoma (PAAD) and stomach adenocarcinoma (STAD).

Presence of tumor-infiltrating lymphocytes is an independent predictor of survival in cancers. Therefore, we used the TIMER database to explore the relationship between YAP1 expression and the degree of immune cell infiltration in 39 tumor types (Supplementary Figure S1). Our findings suggested that YAP1 expression is significantly correlated with the tumor purity in 13 cancer types, B cell infiltration in 17 cancer types, CD4^+^ T cell infiltration in 25 cancer types, CD8^+^ T cell infiltration in 26 cancer types, macrophage infiltration in 27 cancer types, neutrophil infiltration in 26 cancer types, and DC infiltration in 24 cancer types. In PAAD, the expression of YAP1 was significantly related to levels of B cells (*R* = 0.297, *p* = 7.95e−05), CD8^+^ T cells (*R* = 0.625, *p =* 6.21e−20), macrophages (*R* = 0.485, *p* = 1.71e−11), neutrophils (*R* = 0.461 *p* = 2.21e−10), and DCs (*R* = 0.493, *p* = 7.80e−12), whereas there were no correlations with CD4^+^ T cells and tumor purity (Figure 7A). We did not detect significant associations between YAP1 levels and tumor purity or B cell, CD8^+^ T cell, neutrophil, and DC infiltration in STAD (Figure 7B). By generating KM plots using the TIMER database, we further explored the correlation between YAP1 expression and immune cell infiltration in PAAD and STAD. We found that mRNA expression of YAP1 was significantly correlated with prognosis in PAAD (*p* = 0.003) (Figure 8A) and with prognosis and macrophage infiltration in STAD (*p* = 0.004) (Figure 8B). These results indicate that YAP1 plays an important role in the regulation of immune cell infiltration in pancreatic cancer, with a particularly strong role in the infiltration of macrophages, CD8^+^ T cells, neutrophils, and DCs.

### Relationships Between YAP1 and Immune Marker Expression

**FIGURE 9:**
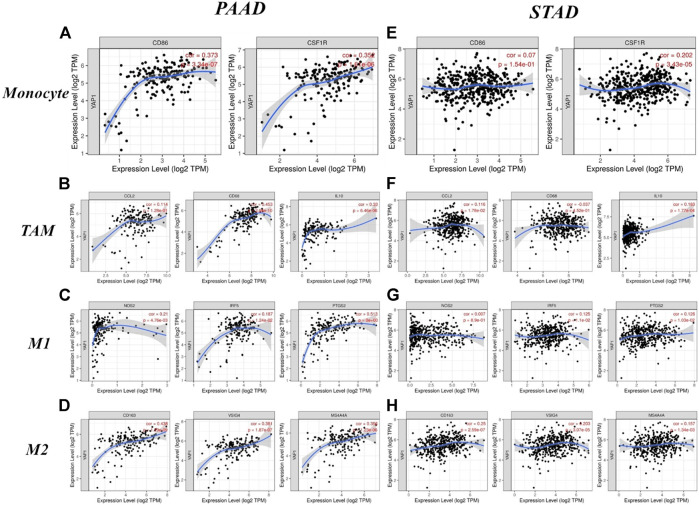
Correlations between YAP1 expression and macrophage polarization in pancreatic adenocarcinoma (PAAD) and stomach adenocarcinoma (STAD). Markers include CD86 and CSF1R for monocytes; CCL2, CD68, and IL10 for tumor-associated macrophages (TAMs); NOS2, IRF5, and PTGS2 for M1 macrophages; and CD163, VSIG4, and MS4A4A for M2 macrophages.

Next, we used the TIMER and GEPIA databases, based on immunological markers sets in PAAD, to explore the relationship between the expression of YAP1 and immune cell infiltration, with STAD as the control group. In addition, we evaluated the relationship between YAP1 expression and several immunological marker subsets, including markers of total T cells, CD8^+^ T cells, B cells, tumor-associated macrophages (TAMs), monocytes, M1 and M2 macrophages, NK cells, neutrophils, DCs, Tfh cells, Th1 cells, Th2 cells, Tregs, Th17 cells, and exhausted T cells. These results were adjusted based on tumor purity. We detected a significant association between YAP1 expression and markers of total T cells (CD3E and CD2), CD8^+^ T (CD8A and CD8B), TAMs (CD68 and IL10), monocytes (CD86 and CD115), M1 macrophages (INOS, IRF5, and COX2), M2 macrophages (CD163, VSIG4, and MS4A4A), NK cells (KIR2DL4), neutrophils (CD11b), DCs (HLA-DPB1, HLA-DQB1, HLA-DRA, HLA-DRA, HLA-DRA1, BDCA-4, and CD11C), Tfh (BCL6), Th1 (STAT1 and IFN-γ), Th2 (GATA3, STAT6, and STAT5A), Th17 (STAT3), and Tregs (FOXP3, CCR8, STAT5B, and TGFβ1) in PAAD (Table 2). Conversely, the expression level of YAP1 was associated with only 18 of the markers in STAD (Table 2). Our findings suggest that YAP1 expression had significant correlations with the levels of majority of the markers of TAMs, monocytes, and M1 and M2 macrophages in PAAD (Table 2). Remarkably, YAP1 expression was closely related to levels of CD86 and CD115 (monocyte markers); CD68 and IL10 (TAM markers); COX2 (M1 macrophage marker); and CD163, VSIG4, and MS4A4A (M2 macrophage markers) (*p* < 0.0001; Figures 9A–D).

**TABLE 2:** Correlations between YAP1 and markers of immune cells in TIMER.

Description	Gene markers	PAAD				STAD			
		None		Purity		None		Purity	
		Cor	*p*	Cor	*p*	Cor	*p*	Cor	*p*
CD8+T cell	CD8A	0.254	**	0.211	*	0.12	0.0143	0.114	0.0261
	CD8B	0.192	*	0.142	0.0644	0.142	3.66e−03	0.134	*
T Cell (general)	CD3D	0.166	0.0266	0.109	0.158	−0.01	0.842	−0.003	0.957
	CD3E	0.198	*	0.141	0.0657	0.018	0.708	0.033	0.525
	CD2	0.207	*	0.146	0.0538	0.078	0.114	0.091	0.0767
B Cell	CD19	0.106	0.157	0.067	0.383	−0.016	0.751	0.001	0.991
	CD79A	0.133	0.0761	0.086	0.265	−0.1	0.042	−0.09	0.0805
Monocyte	CD86	0.373	***	0.327	***	0.07	0.154	0.083	0.106
	CD115 (CSF1R)	0.352	***	0.297	***	0.202	3.43e−05	0.209	***
TAM	CCL2	0.114	0.128	0.081	0.292	0.116	0.0178	0.132	*
	CD68	0.453	***	0.415	***	−0.037	0.452	−0.044	0.39
	IL10	0.33	***	0.299	***	0.183	1.77e−04	0.213	***
M1 macrophage	INOS (NOS2)	0.21	*	0.004	*	0.007	0.89	0.021	0.677
	IRF5	0.187	*	0.172	0.0248	0.125	0.011	0.124	0.0153
	COX2 (PTGS2)	0.513	***	0.529	***	0.126	0.0103	0.131	0.0105
M2 macrophage	CD163	0.438	***	0.39	***	0.25	***	0.259	***
	VSIG4	0.381	***	0.324	1.51e−05	0.203	3.07e−05	0.218	***
	MS4A4A	0.356	***	0.303	5.51e−05	0.157	1.34e−03	0.163	*
Neutrophils	CD66b (CEACAM8)	0.155	0.0387	0.116	0.131	0.037	0.449	0.041	0.426
	CD11b (ITGAM)	0.337	***	0.279	**	0.09	0.0656	0.095	0.0644
	CCR7	0.089	0.235	0.045	0.558	0.067	0.172	0.093	0.0705
Natural killer cell	KIR2DL1	0.124	0.0982	0.13	0.0902	0.121	0.0139	0.108	0.035
	KIR2DL3	0.176	0.0182	0.17	0.026	0.082	0.0969	0.072	0.16
	KIR2DL4	0.206	*	0.22	*	−0.047	0.339	−0.063	0.222
	KIR3DL1	0.05	0.507	0.043	0.575	0.089	0.0697	0.071	0.167
	KIR3DL2	0.136	0.0695	0.105	0.173	0.064	0.196	0.063	0.222
	KIR3DL3	0.151	0.044	0.129	0.0927	−0.089	0.0686	−0.108	0.0359
	KIR2DS4	0.022	0.0775	0.035	0.648	0.012	0.802	0.01	8.48e−01
Dendritic cell	HLA-DPB1	0.23	*	0.174	2.28e−02	−0.021	0.673	−0.008	0.879
	HLA-DQB1	0.235	*	0.191	0.0124	−0.026	0.597	−0.007	0.895
	HLA-DRA	0.367	***	0.323	***	0.005	0.925	0.021	0.688
	HLA-DPA1	0.345	***	0.305	***	−0.006	0.906	0.009	0.856
	BCDA-1 (CD1C)	0.139	0.0632	0.097	0.205	0.03	0.539	0.061	0.235
	BDCA-4 (NRP1)	0.604	***	0.582	***	0.341	***	0.355	***
	CD11c (ITGAX)	0.224	*	0.159	0.0373	0.113	0.0216	0.12	0.0191
Th1	T-bet (TBX21)	0.117	0.119	0.079	0.302	0.096	0.0509	0.114	0.0266
	STAT4	0.035	0.644	0.044	0.569	0.132	*	0.154	*
	STAT1	0.545	***	0.521	***	0.289	***	0.281	***
	IFN-γ (IFNG)	0.196	*	0.178	1.98e−02	0.074	0.13	0.071	0.166
	TNF-α (TNF)	0.092	0.221	0.075	0.33	−0.061	0.215	−0.037	0.47
Th2	GATA3	0.258	**	0.248	*	0.048	0.326	0.058	0.257
	STAT6	0.433	***	0.428	***	0.297	***	0.278	***
	STAT5A	0.295	***	0.259	**	0.312	***	0.314	***
	IL13	−0.04	0.595	−0.023	0.763	0.034	0.485	0.05	0.332
Tfh	BCL6	0.606	***	0.593	***	0.451	***	0.474	***
	IL21	0.064	0.396	0.059	0.44	0.021	0.67	0.019	0.717
Th17	STAT3	0.585	***	0.567	***	0.447	***	0.451	***
	IL17A	0.1	0.184	0.078	0.311	−0.075	0.128	−0.059	0.249
Treg	FOXP3	0.299	***	0.259	**	0.055	0.26	0.061	0.234
	CCR8	0.446	***	0.415	***	0.199	***	0.195	**
	STAT5B	0.313	***	0.376	***	0.472	***	0.486	***
	TGFβ (TGFB1)	0.258	**	0.224	*	0.243	***	0.265	***
T Cell exhaustion	PD-1 (PDCD1)	0.162	0.0299	0.104	0.178	0.087	0.0782	0.09	0.0785
	CTLA4	0.212	*	0.159	0.0382	0.146	*	0.161	*
	LAG3	0.089	0.238	0.064	0.408	0.023	0.64	0.01	0.846
	TIM-3 (HAVCR2)	0.35	***	0.297	***	0.115	1.95e−02	0.118	0.0214
	GZMB	0.23	*	0.187	0.0144	0.031	0.527	0.027	0.599

Cor, Spearman correlation coefficient *R*; None, correlation without adjustment. Purity, correlation adjusted by purity. *p < 0 0.01; **p < 0.001; ***p < 0.0001.

Abbreviations: PAAD, pancreatic adenocarcinoma; STAD, stomach adenocarcinoma; TAM, tumor-correlated macrophage; Tfh, follicular helper T cell; Th, T helper cell; Treg, regulatory T cell.

We also explored relationships between YAP1 expression and the expression levels of monocyte, M1 and M2 macrophage, and TAM markers in PAAD and STAD using the GEPIA database. The correlations between PAAD and monocyte and TAM markers were similar to those in the TIMER analysis (Table 3). These findings suggested that YAP1 regulates macrophage polarization in PAAD. Additionally, in PAAD, high YAP1 expression was associated with high levels of DC infiltration, and DC markers, such as HLA-DRA, HLA-DPA1, BDCA-4 and CD11c, were also significantly associated with YAP1 expression. Our findings further indicated a significant correlation between YAP1 and DC infiltration. Furthermore, for Treg cells and exhausted T cells, YAP1 expression was significantly associated with FOXP3, STAT5B, CCR8, CTLA4, TGFβ, TIM-3, and GZMB in PAAD (Table 2). DCs promoted tumor metastasis by reducing CD8^+^ T cells and increasing Treg cell cytotoxicity (Gebhardt and Harvey, 2016; Ni et al., 2018; Meng et al., 2020). It is not clear whether YAP1 is a key factor in tumor metastasis and DC infiltration. FOXP3 has a crucial role in Treg cells, suppressing the effect of cytotoxic T cells on tumor cells (Kim et al., 2018; He et al., 2019). TIM-3 is a key factor in the regulation of T cell exhaustion. As evidenced by the significant association between YAP1 expression and both FOXP3 and TIM-3, high YAP1 expression contributes to TIM-3-mediated T cell exhaustion. These results confirmed that the expression of YAP1 was significantly related to infiltrating immune cells in PAAD and played a significant role in immune escape in the pancreatic cancer microenvironment.

**TABLE 3:** Correlations between YAP1 expression and markers of monocytes and macrophages in GEPIA Description Gene markers PAAD STAD.

Description	Gene markers	Tumor *Cor p*		Normal *Cor p*		Tumor *Cor p*		Normal *Cor p*	
Monocyte	CD86	0.41	***	0.42	0.6	0.1	0.04	−0.54	**
	CD115 (CSF1R)	0.39	***	0.2	0.92	0.29	3.3e−09	−0.17	0.31
TAM	CCL2	0.15	0.045	0.4	0.75	0.14	*	0.2	0.23
	CD68	0.55	***	0.8	0.33	0.1	0.037	−0.35	0.037
	IL10	0.37	***	0.8	0.33	0.27	***	−0.35	0.039
M1 macrophage	INOS (NOS2)	0.25	***	0.4	0.75	0.032	0.52	−0.12	0.47
	IRF5	0.21	*	−1	0.083	0.24	***	0.1	0.56
	COX2 (PTGS2)	0.5	***	0.4	0.75	0.17	***	0.43	*
M2 macrophage	CD163	0.37	***	0.4	0.75	0.17	***	0.32	0.061
	VSIG4	0.38	***	0.8	0.33	0.25	***	−0.041	0.81
	MS4A4A	0.39	***	0.8	0.33	0.2	***	−0.001	1

PAAD, pancreatic adenocarcinoma; STAD, stomach adenocarcinoma; TAM, Tumor-associated macrophages. Tumor, tumor tissue in TCGA. Normal, normal tissue in TCGA. Cor, Spearman correlation coefficient *R*, *p < 0.01; **p < 0.001; ***p < 0.0001.

## Discussion

YAP1 is a downstream effector of the Hippo signaling pathway (Wang et al., 2016; Ma et al., 2020). It is negatively regulated by upstream factors in the Hippo pathway; when this pathway is activated, it is exported to the cytoplasm and degraded (Wang et al., 2017; Liu et al., 2019). In normal cells, Hippo/YAP is a crucial determinant of organ size (Wang et al., 2017; Elster et al., 2018). YAP1 is overexpressed in many cancers, such as colorectal, lung, liver, ovarian, and prostate cancers, and promotes tumor formation and development (Zhou et al., 2018; Nguyen and Yi, 2019; White et al., 2019; Yang et al., 2020). There is accumulating evidence that YAP1 facilitates the immunosuppressive tumor microenvironment, affecting myeloid-derived suppressor cells, macrophages, and regulatory T-cells (Shibata et al., 2018; Taha et al., 2018; White et al., 2019). However, the underlying mechanisms by which YAP1 contributes to tumor immunity is not clearly established (Hong et al., 2018; Zhang et al., 2018; Wang et al., 2020; Zhang et al., 2020).

In this study, we found that YAP1 expression is associated with prognosis in several types of cancer. In particular, our analyses showed that increased YAP1 expression is associated with a poorer prognosis in PAAD. Furthermore, expression levels of YAP1 were significantly related to levels of immune cell infiltration and diverse immune marker sets in PAAD. Thus, these results suggest that YAP1 contributes to the immune response in PAAD and may be a novel prognostic biomarker.

We examined the expression levels of YAP1 in multiple tumors and corresponding normal tissues using datasets from Oncomine, TCGA in TIMER, and GEPIA databases. YAP1 was differentially expressed between tumor tissues and normal tissues in multiple cancer types. YAP1 expression was upregulated or downregulated in various cancers (Figures 1–3). The heterogeneity of YAP1 expression among cancer types and databases may be attributed to differences in data collection methods and analytical approaches. Nevertheless, we consistently observed a correlation between higher expression of YAP1 and a poor prognosis in PAAD across these databases.

We selected several cancers with the most obvious expression differences between tumor tissues and normal adjacent tissues (breast, pancreatic, colorectal, esophageal, gastric, and lung cancers) and explored the critical role of YAP1 in patient outcomes. Using the KM plotter, GEPIA, and TCGA databases, we found that high YAP1 expression was significantly related to a poorer prognosis in pancreatic cancer (Figures 4–6). These findings suggest that YAP1 is a novel prognostic biomarker for pancreatic cancer.

Another important aspect of this research was the finding that the expression of YAP1 is significantly correlated with diverse immune cell infiltration levels in multiple cancer types, especially in pancreatic cancer. We detected a strong positive association between the expression level of YAP1 and infiltration level of CD8^+^ T cells, moderate positive associations between YAP1 expression and the infiltration of macrophages, neutrophils, and DCs, and significant positive associations between the infiltration of B cells and YAP1 expression in PAAD, with no relationships between YAP1 and CD4+T cells and tumor purity (Figure 7A). These results indicate that YAP1 plays an important role in the regulation of immune cell infiltration in pancreatic cancer, with particularly strong effects on CD8^+^ T cells, macrophages, neutrophils, and DCs infiltration.

Furthermore, to investigate the role of YAP1 in the regulation of tumor immunology in PAAD, we analyzed the relationships between YAP1 expression and marker genes of immune cells. Our results suggested that markers of M1 macrophages (such as NOS2 and IRF5) showed weak associations with YAP1 expression, and PTGS2 showed a moderate relationship with YAP1 expression in PAAD (Tables 2 and 3). M2 macrophage markers (including CD163, MS4A4A, and VSIG4) showed moderate and strong correlations with the expression levels of YAP1 in PAAD (Tables 2 and 3). These findings revealed the potential contribution of YAP1 to TAM polarization. Our findings also suggest that YAP1 may function in the activation of Tregs and induction of T cell exhaustion. Gene markers of Treg and T cell exhaustion, including FOXP3, STAT5B, CCR8, TGFβ, CTLA4, TIM-3, and GZMB, showed moderate or weak correlations with YAP1 expression in PAAD (Table 3). In particular, as a key surface protein in T cell exhaustion, TIM-3 and YAP1 expression levels were closely related in PAAD. Accordingly, YAP1 may suppress T cell-mediated immunity by promoting Treg responses. Moreover, our results demonstrated the relationship between YAP1 expression and T helper cells, such as Th1, Th2, Tfh, and Th17. We found that Th1 markers (STAT1 and IFN-γ), Th2 markers (GATA3, STAT6, and STAT5A), a Tfh marker (BCL6), and a Th17 marker (STAT3) were significantly positively correlated with YAP1 in PAAD. YAP1 may therefore regulate T cell responses in PAAD. Together, these findings suggested that YAP1 is a crucial factor for the recruitment and regulation of infiltrating immune cells in PAAD.

In summary, YAP1 can be a valuable prognostic biomarker as well as a crucial regulator of immune cell infiltration in patients with pancreatic cancer.

## Data Availability

The original contributions presented in the study are included in the article/Supplementary Material, further inquiries can be directed to the corresponding author.

## Glossary

YAP1: yes-associated protein-1
GEPIA: gene expression profiling interactive analysis
LOGpc: long-term outcome and gene expression profiling database of pan-cancers
TCGA: the cancer genome atlas
PC: pancreatic cancer
RT: chemotherapy and radiotherapy
EMT: epithelial–mesenchymal transition
NSCLC: non-small cell lung cancer
MPM: malignant pleural mesothelioma
TIMER: tumor immune estimation resource
ONCOMINE: online cancer microarray database
KM plotter: Kaplan–Meier plotter
BLCA: bladder urothelial carcinoma: bladder urothelial carcinoma
BRCA: breast invasive carcinoma
KICH: kidney chromophobe
KIRC: kidney renal clear cell carcinoma
KIRP: kidney renal papillary cell carcinoma
LIHC: liver hepatocellular carcinoma
LUAD: lung adenocarcinoma
LUSC: lung squamous cell carcinoma
PRAD: prostate adenocarcinoma
UCEC: uterine corpus endometrial carcinoma: uterine corpus endometrial carcinoma
CHOL: cholangial carcinoma
COAD: colon adenocarcinoma
STAD: stomach adenocarcinoma
DLBC: lymphoid neoplasm diffuse large B-cell lymphoma
GBM: glioblastoma multiforme
PAAD: pancreatic adenocarcinoma
THYM: thymoma
ACC: adrenocortical carcinoma
BLCA: bladder urothelial carcinoma: bladder urothelial carcinoma
PCPG: pheochromocytoma and paraganglioma
UCEC: uterine corpus endometrial carcinoma: uterine corpus endometrial carcinoma
UCS: uterine carcinosarcoma
OS: overall survival
DFS: disease-free survival
RFS: relapse-free survival
DSS: disease-specific survival
DMFS: distant metastasis-free survival
FP: first progression
TAM: tumor-associated macrophages
DCs: dendritic cells
