# Supplementary material for: YAP1 is a Prognostic Biomarker and Correlated with Immune Cell Infiltration in Pancreatic Cancer
Source: Front Mol Biosci. 2021 Jun 2;8:625731. doi: 10.3389/fmolb.2021.625731 (PMC8207136; doi:10.3389/fmolb.2021.625731)
Supplement: Supplementary file 1 [file DataSheet1.docx]

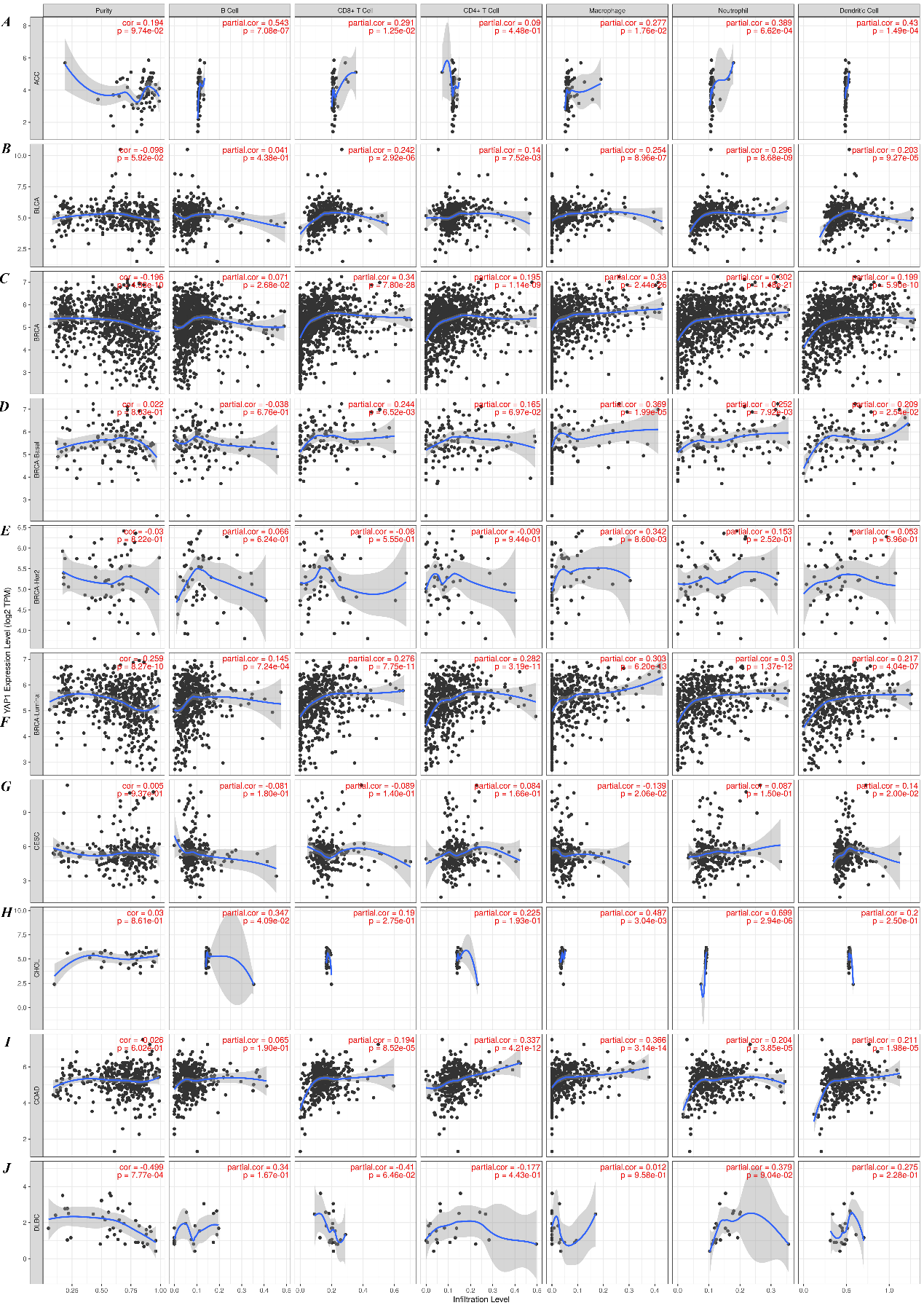

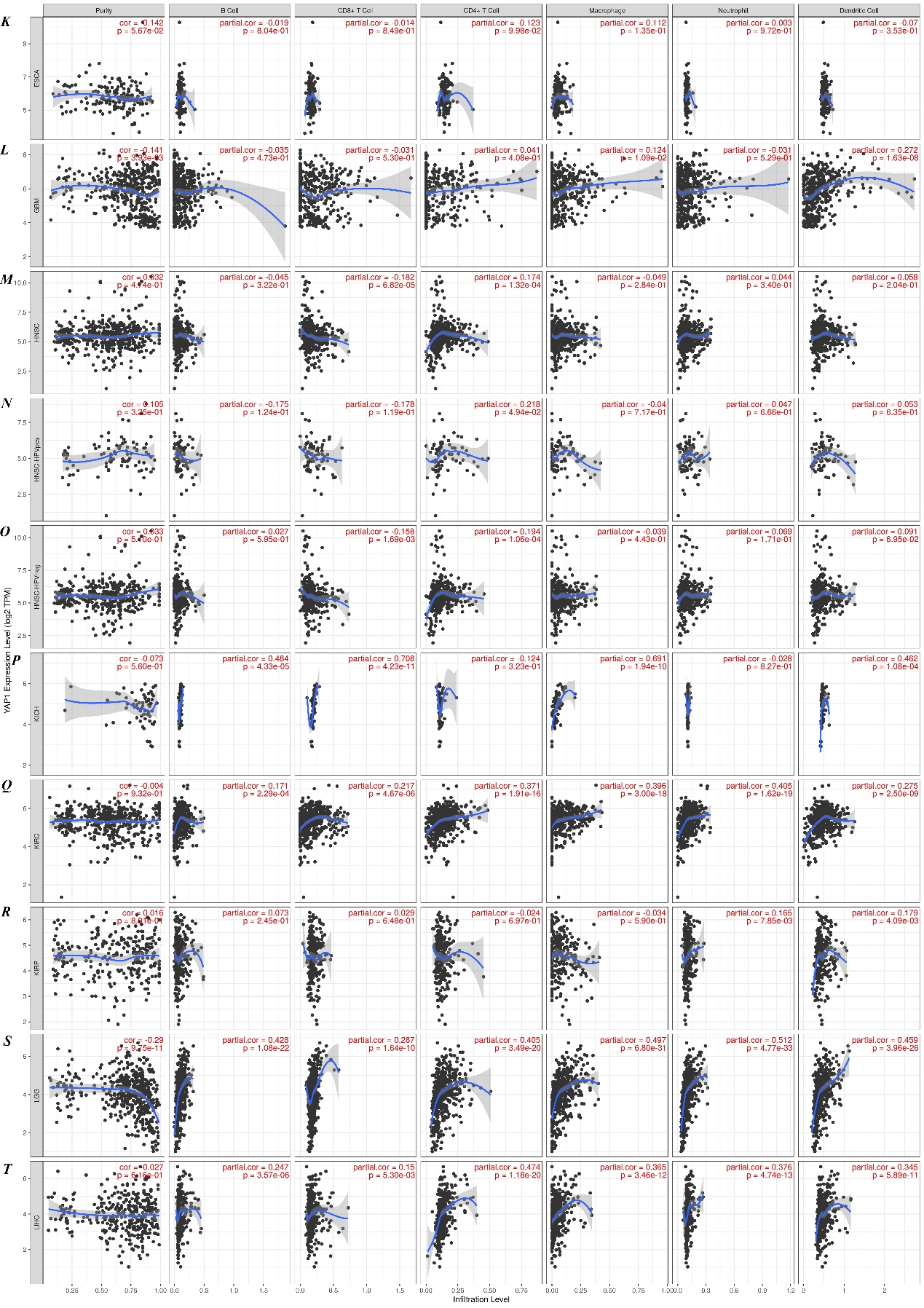

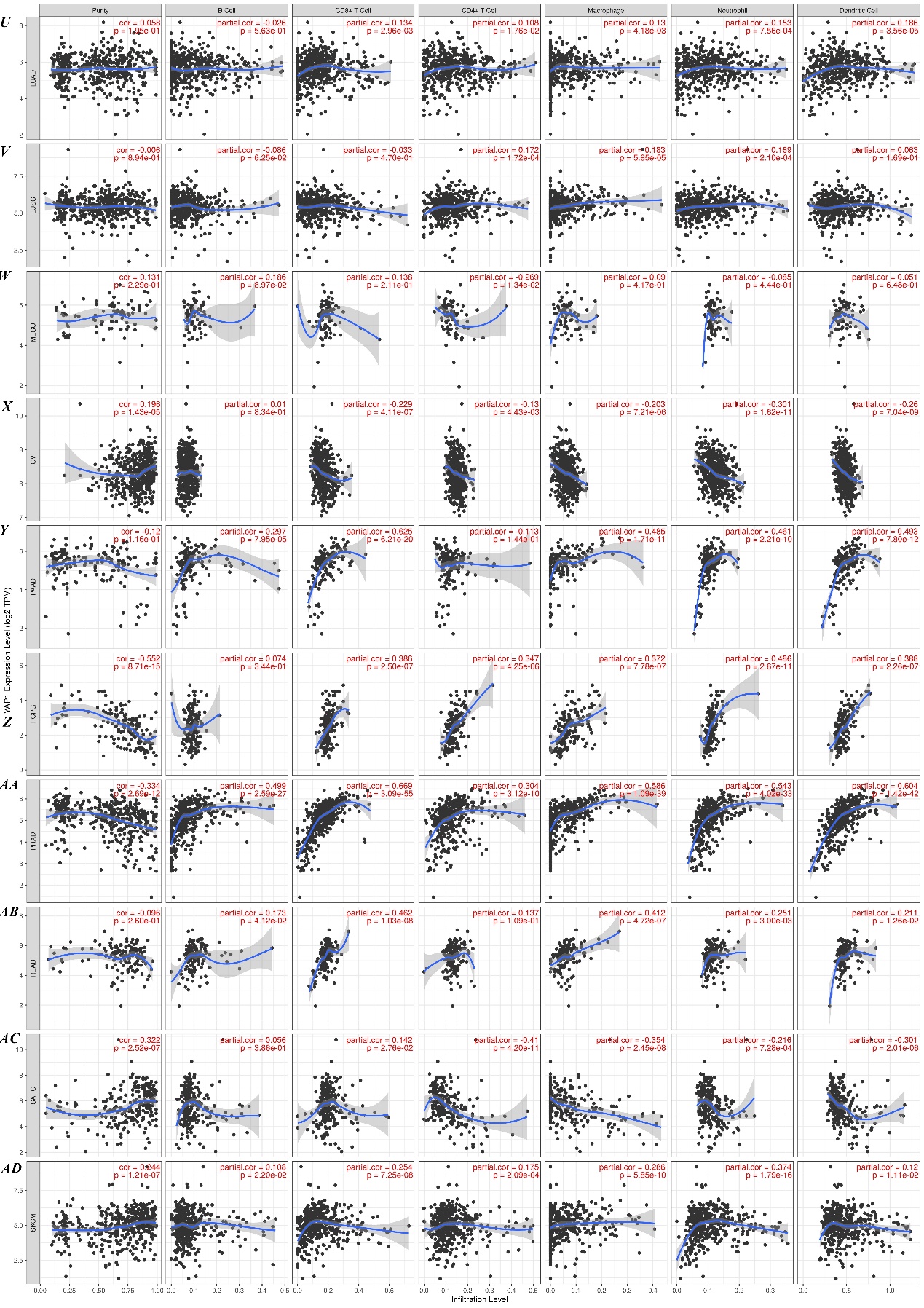

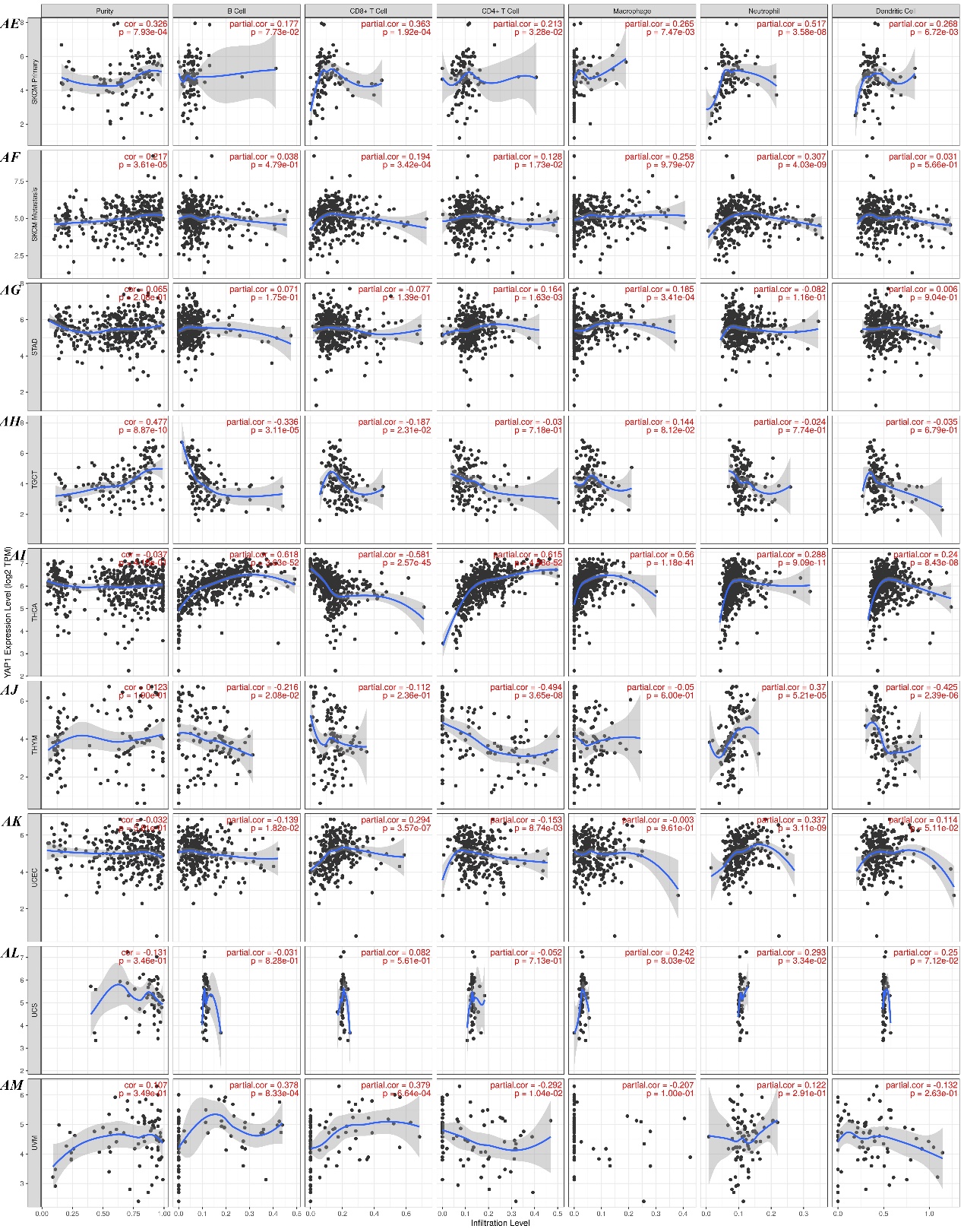
 Supplementary Figure 1. Correlation of YAP1 expression with immune infiltration levels in Adrenocortical carcinoma(ACC) (A) , Bladder Urothelial Carcinoma (BLCA) (B), Breast invasive carcinoma (BRCA) (C), Breast invasive carcinoma-Basal (BRCA-Basal) (D), Breast invasive carcinoma-Her2 (BRCA-Her2) (E), Breast invasive carcinoma-Luminal (BRCA- Luminal) (F), Cervical squamous cell carcinoma and endocervical adenocarcinoma(CESC) (G), Cholangio carcinoma(CHOL) (H), colon adenocarcinoma (COAD)(I), Lymphoid Neoplasm Diffuse Large B-cell Lymphoma(DLBC) (J), Esophageal carcinoma(ESCA) (K), Glioblastoma multiforme(GBM) (L) ,Head and Neck squamous cell carcinoma(HNSC) (M), Head and Neck squamous cell carcinoma HPVpos(HNSC-HPVpos) (N) , Head and Neck squamous cell carcinoma-HPVneg(HNSC-HPVneg) (O), Kidney Chromophobe(KICH) (P), Kidney renal clear cell carcinoma(KIRC) (Q), Kidney renal papillary cell carcinoma(KIRP) (R), Brain Lower Grade Glioma(LGG) (S), Liver hepatocellular carcinoma(LIHC) (T), Lung adenocarcinoma(LUAD) (U), lung squamous cell carcinoma(LUSC)(V), Mesothelioma(MESO) (W), Ovarian serous cystadenocarcinoma(OV) (X), Pancreatic adenocarcinoma(PAAD) (Y), Pheochromocytoma and Paraganglioma(PCPG) (Z), Prostate adenocarcinoma(PRAD) (AA), Rectum adenocarcinoma(READ) (AB), Sarcoma(SARC) (AC), Skin Cutaneous Melanoma(SKCM) (AD), Skin Cutaneous Melanoma-Primary(SKCM- Primary) (AE), Skin Cutaneous Melanoma-Metastasis(SKCM- Metastasis) (AF), stomach adenocarcinoma(STAD)(AG), Testicular Germ Cell Tumors(TGCT) (AH), Thyroid carcinoma(THCA) (AI), Thymoma(THYM) (AJ), Uterine Corpus Endometrial Carcinoma(UCEC) (AK), Uterine Carcinosarcoma(UCS) (AL), Uveal Melanoma(UVM) (AM)
